# Revealing charge heterogeneity of stressed trastuzumab at the subunit level

**DOI:** 10.1007/s00216-023-04547-4

**Published:** 2023-01-25

**Authors:** Baubek Spanov, Bas Baartmans, Oladapo Olaleye, Simone Nicolardi, Natalia Govorukhina, Manfred Wuhrer, Nico C. van de Merbel, Rainer Bischoff

**Affiliations:** 1 Department of Analytical Biochemistry, Groningen Research Institute of Pharmacy, University of Groningen, A Deusinglaan 1, 9713 AV Groningen, The Netherlands; 2 Center for Proteomics and Metabolomics, Leiden University Medical Center, 2333 ZA Leiden, The Netherlands; 3 Bioanalytical Laboratory, ICON, Amerikaweg 18, 9407 TK Assen, The Netherlands

**Keywords:** Cation-exchange chromatography, pH gradient, Trastuzumab, Charge variant

## Abstract

Trastuzumab is known to be heterogeneous in terms of charge. Stressing trastuzumab under physiological conditions (pH 7.4 and 37 °C) increases charge heterogeneity further. Separation of charge variants of stressed trastuzumab at the intact protein level is challenging due to increasing complexity making it difficult to obtain pure charge variants for further characterization. Here we report an approach for revealing charge heterogeneity of stressed trastuzumab at the subunit level by pH gradient cation-exchange chromatography. Trastuzumab subunits were generated after limited proteolytic cleavage with papain, IdeS, and GingisKHAN®. The basic pI of Fab and F(ab)_2_ fragments allowed to use the same pH gradient for intact protein and subunit level analysis. Baseline separation of Fab subunits was obtained after GingisKHAN® and papain digestion and the corresponding modifications were determined by LC–MS/MS peptide mapping and middle-down MALDI-ISD FT-ICR MS. The described approach allows a comprehensive charge variant analysis of therapeutic antibodies that have two or more modification sites in the Fab region.

**Supplementary Information:**

The online version contains supplementary material available at 10.1007/s00216-023-04547-4.

## Introduction

Monoclonal antibodies (mAbs) are inherently heterogeneous [1]. N-glycosylation heterogeneity may be introduced during expression in cell lines and results in the formation of glycosylated variants (glycoforms) [1, 2]. Heterogeneity may also be introduced during manufacturing or due to degradation during storage [1]. Incomplete disulfide bond formation, deamidation, oxidation, N-terminal glutamine cyclization, and isomerization are examples of non-enzymatic modifications. Such heterogeneity may impact the stability, safety, and activity of therapeutic mAbs.

Charge-related heterogeneities of mAbs have often been assigned as critical quality attributes (CQAs) [3, 4]. Modifications such as deamidation, glycation, sialyation, and C-terminal lysine truncation are contributors to charge heterogeneity of mAbs. In order to demonstrate product consistency and shelf-life stability, charge heterogeneity of therapeutic mAbs must be monitored during development and production. To ensure the safety and efficacy of the drug, and to gain regulatory approval, charge variants of mAbs need to be detected, quantified, and characterized. Forced degradation studies are often applied to monitor the long-term stability of mAbs and detect hotspots for modifications by intentionally degrading them under different stress conditions. Stressing mAbs at basic pH and elevated temperature is the most commonly used approach to induce modifications that contribute to charge heterogeneity [5, 6].

Ion-exchange chromatography and electrophoretic separation methods have been widely used to separate charge variants of mAbs [7–10]. Other chromatographic approaches may also be employed for this purpose, such as hydrophobic interaction chromatography [11–14] and displacement chromatography [15, 16]. Cation-exchange chromatography (CEX) is the method of choice due to the basic pI of most mAbs [8, 17–29]. Charge variants of mAbs can be eluted from the cation-exchange column either by salt [17, 19, 27] or pH gradients [18, 20–27]. Several studies have reported that pH gradient separation outperforms salt gradients in terms of resolution [22, 28]. pH gradient separation is also attractive because of the possibility of coupling to a mass spectrometer when using volatile buffers [21, 24–26]. However, volatile pH gradient buffers are known to suffer from non-linear pH change over the gradient, which is one of the key parameters for achieving superior separation.

Trastuzumab is a humanized monoclonal antibody used in the clinic for the treatment of patients with HER2-positive breast cancer. Patients receive a fresh dose of trastuzumab every 3 weeks. Therefore, we stressed trastuzumab under physiological conditions (pH 7.4, 37 °C) for 3 weeks to see changes in charge variant composition and to mimic what might be happening in vivo. We have previously reported the application of pH gradient CEX for the separation of charge variants of trastuzumab upon stressing [20] and to study in vivo biotransformation [30]. Charge variant profiles of trastuzumab enriched from patient samples were highly similar to the charge variant profile of 3-week stressed samples in vitro. Although the use of a pH gradient resulted in high-resolution separations, samples stressed for 3 weeks at pH 7.4 and 37 °C showed very complex chromatograms with overlapping peaks. For comprehensive analysis of charge heterogeneity, we therefore looked for options to reduce the complexity of the cation-exchange profile of stressed trastuzumab. For this purpose, stressed trastuzumab was digested into smaller subunits with papain, IdeS, and GingisKHAN. Charge variants separated at the Fab level were characterized by peptide mapping and middle-down matrix-assisted laser desorption ionization in-source decay (MALDI ISD) Fourier transform ion cyclotron resonance (FT-ICR) mass spectrometry (MS).

## Materials and chemicals

Trastuzumab (Herceptin®, Lot N3024H10) was obtained from Roche (Grenzach-Wyhlen, Germany). IdeS (FabRICATOR®, cat# A0-FR1-008) and GingisKHAN® (cat# B0-GKH-020) were purchased from Genovis (Lund, Sweden). Papain was obtained from FUJIFILM Wako Chemicals (cat# 166–00,171). Trypsin/Lys-C Mix, Mass Spec Grade, (cat# V5073) was purchased from Promega (Madison, WI, USA). Difluoroacetic acid (DFA, cat# 162,120,025) was obtained from Acros Organics (Fair Lawn, NJ, USA). 2-(N-morpholino)ethanesulfonic acid, 4-morpholineethanesulfonic acid monohydrate (MES monohydrate, cat# 69,892), 4-(2-hydroxyethyl)piperazine-1-ethanesulfonic acid (HEPES, cat# H4034), N,N-bis(2-hydroxyethyl)glycine (bicine, cat# B3876), 3-(cyclohexylamino)-2-hydroxy-1-propanesulfonic acid (CAPSO, cat# C2278), 3-(cyclohexylamino)-1-propanesulfonic acid (CAPS, cat# C6070), sodium chloride (cat# 746,398), DL-dithiothreitol (DTT, cat# D0632), iodoacetamide (IAA, cat# 16,125), formic acid (cat# 695,076), sodium deoxycholate (SDC, cat# 30,970), and sodium dihydrogen phosphate monohydrate (cat# 1,063,460,500) were obtained from Sigma-Aldrich (St. Louis, Missouri, USA). PBS was purchased from Thermo Fisher Scientific (cat# 14,200–067, Life Technologies Limited, Paisley, UK).

### Forced degradation study

Herceptin (trastuzumab) was purchased as a lyophilized powder that contains 150 mg of trastuzumab, 3.4 mg L-histidine HCl monohydrate, 2.2 mg L-histidine, and some other components in the vial. It was dissolved with 5 mL of Milli-Q water to obtain a concentration of 30 mg/mL. The reconstituted solution had a pH of approximately 6 due to the presence of L-histidine in the formulation. The solution was stored at 4 °C. We did not observe significant changes in the charge variant profile of trastuzumab upon storage.

A stock solution of trastuzumab at a concentration of 30 mg/mL was diluted to 5 mg/mL with PBS, pH 7.4. 1500 µL of 5 mg/mL trastuzumab was stressed by incubation for 3 weeks at 37 °C, after which the pH of the diluted samples was adjusted to 6.5 with 20 mM MES buffer to prevent further modifications.

### Generation of subunits

Heavy and light chains of trastuzumab were generated by inter-chain disulfide bond reduction. A 3 weeks stressed sample was reduced in 100 mM phosphate buffer pH 7.4 in the presence of 10 mM DTT (final concentration) for 20 min at 37 °C. The sample was directly injected into the cation-exchange column without quenching DTT.

IdeS digestion of 3 weeks stressed trastuzumab was performed for 45 min in 100 mM phosphate buffer pH 7.4 and 37 °C. The protein to enzyme ratio was set to 1 µg:1U according to the manufacturer’s recommendations.

GingisKHAN digestion of 3 weeks stressed trastuzumab was performed for 2 h in 100 mM phosphate buffer pH 7.4 and 37 °C. The protein to enzyme ratio was set to 1 µg:1U according to the manufacturer’s recommendations.

Papain digestion of 3 weeks stressed trastuzumab was performed in 100 mM phosphate buffer at pH 7 overnight (18 h) at 37 °C in the presence of 5 mM cysteine and 2 mM EDTA. The protein to papain ratio was set to 25:1 (w/w).

### pH gradient CEX

pH gradient CEX of charge variants was performed on an Agilent 1200 series HPLC system with a MabPac SCX-10 column (2.1 × 250 mm, 5 µm) that was coupled to a G4212B Diode Array Detector. Buffer A (5.5 mM HEPES, 4.2 mM Bicine, 9.5 mM CAPSO, 0.8 mM CAPS) had a pH of 8, and buffer B (10.5 mM Bicine, 2.5 mM CAPSO, 7.0 mM CAPS) had a pH of 10.5, as described previously [20, 22]. A 10-column volume gradient (58 min) was applied at a flow rate of 0.15 mL/min, with the composition of buffer B varying from 0 to 60%. The separation was performed at room temperature. UV absorbance was measured at 280 nm. 80 µg of antibody/antibody digest on column was injected in all pH gradient CEX experiments.

Fab fractions from the cation-exchange column were collected in Protein LoBind 96-well plates filled with 300 mM MES pH 6. The manual fraction collection was performed in multiple consecutive runs based on the retention time and peak width. Fractions were concentrated with Amicon Ultra-4 10 kDa cut-off centrifugal units. Centrifugation was done according to the manufacturer’s recommendations at 3000 × *g* using a swinging bucket rotor at 20 °C for 15 min. After concentrating the fractions, the buffer exchange was performed with 5 mM MES pH 6. The protein concentration in fractions was determined on a NanoPhotometer® N120 (Implen GmbH, Munich, Germany) at 280 nm and varied in the range of 0.4–5 mg/mL depending on the peak intensity in Fig. 3. Samples were stored at 4 °C till further LC–MS/MS and MALDI-ISD FT-ICR MS analysis.

### LC–MS/MS peptide mapping

After CEX and buffer exchange, the Fab fractions were denatured and reduced by mixing 3 µL of the Fab fraction with 5 µL of 0.6% SDC in 50 mM HEPES pH 7 and 2 µL of 25 mM DTT, and the mixture was heated for 30 min at 60 °C in a thermoshaker at 600 rpm. 2 µL of 90 mM IAA was added for alkylation in the dark for 20 min. Samples were digested by adding 3 µL of a trypsin/Lys-C mix at a ratio of 30:1 (protein to enzyme, w/w) for 6 h at 37 °C. SDC was removed by acid precipitation after adding 2 µL of 3% DFA followed by centrifugation at 10,000 rpm for 10 min. 10 µL of the protein digest was injected for peptide mapping.

Peptides were separated on a reversed-phase PepMap C18 column (0.3 × 150 mm, 2 μm, 100 Å, Thermo Fisher Scientific) with a gradient from 2 to 35% B over 65 min, where mobile phase A was 0.1% formic acid in water, and mobile phase B was 0.1% formic acid in acetonitrile. The flow rate was set to 5 μL/min. The column temperature was 40 °C. Samples were stored at 8 °C during the LC–MS/MS analysis.

LC–MS/MS peptide mapping was performed in the data-dependent acquisition mode where an Eksigent NanoLC 425 system with a microflow pump (1–10 μL) was coupled to a TT6600 quadrupole-time-of-flight (QTOF) mass spectrometer with an OptiFlow® source (SCIEX, Toronto, Canada). The OptiFlow® source had the following settings: Ion Source Gas 1 (GS1) 10 psi, Ion Source Gas 2 (GS2) 20 psi, Curtain Gas (CUR) 25 psi, Temperature (TEM) 100 °C, IonSpray Voltage Floating (ISVF) 4500 V, and Declustering Potential (DP) 90 V. Data-dependent acquisition consisted of a cycle where an MS scan from 350 to 2000 m/z was followed by MS/MS of the top five most intense precursor ions detected at a threshold of 100 counts per second. Precursor ions with charge states of 2 to 4 were selected for MS/MS fragmentation with an exclusion window of 4 s after two occurrences. The collision energy for MS/MS fragmentation was calculated based on the m/z value and charge state of the candidate precursor ion with activation of the Rolling Collision Energy option.

Data analysis was performed with the BPV Flex 2.1 and PeakView 2.2 software (SCIEX, Toronto, Canada). The precursor mass error tolerance was set to 15 ppm and the fragment mass error tolerance was set to 0.03 Da. The false discovery rate was set to 1%. Carbamidomethylation was selected as a fixed modification, while methionine oxidation, asparagine deamidation, and N-terminal pyroglutamate formation were selected as variable modifications.

### MALDI-ISD FT-ICR MS

Fab fractions, after CEX and buffer exchange, were desalted using C18-ZipTip (Merck Millipore). Elution from the tip was done with 2 µL of a 50:49.95:0.05 (v/v/v) ACN/water/formic acid solution directly onto a polished-steel MALDI target plate (Bruker Daltonics). 1 µL of a saturated solution of 1,5-diaminonaphthalene (1,5-DAN) in 50:49.95:0.05 (v/v/v) ACN/water/formic acid was added and left to dry at room temperature.

MALDI-ISD FT-ICR MS analysis was performed as previously reported [31, 32]. Briefly, MS measurements were performed on a 15 T solariX XR FT-ICR mass spectrometer (Bruker Daltonics, Bremen, Germany) equipped with a CombiSource and a ParaCell. The MS system was operated using ftmsControl software (Bruker Daltonics). MALDI measurements were performed using a Smartbeam-II laser system (Bruker Daltonics) at a frequency of 500 Hz and 200 laser shots per scan. ISD fragment ions were measured in negative ion mode across the m/z-range 797–7000 with 1 million data points. Internal calibration of the mass spectra was performed using the theoretical m/z-values of c’ fragment ions of the heavy chain calculated using the online tool Protein Prospector (https://prospector.ucsf.edu/prospector/mshome.htm).

## Results

### Ion-exchange profile of intact and reduced trastuzumab

**Fig. 1:**
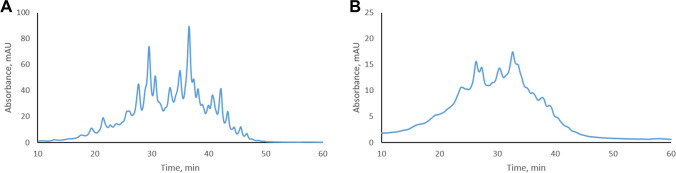
Profile of **A** 3 weeks stressed intact trastuzumab and **B** 3 weeks stressed reduced trastuzumab obtained by pH gradient cation-exchange chromatography. UV absorbance was measured at 280 nm

Generation of free heavy and light chains through inter-chain disulfide bond reduction is the most straightforward approach to obtaining antibody subunits. When 3 weeks stressed trastuzumab was analyzed by pH gradient CEX after the reduction of inter-chain disulfide bonds under native conditions, it showed a complex chromatographic profile that could not be resolved into individual, pure charge variants (Fig. 1). Subsequent alkylation of free SH groups with IAA did not improve the chromatographic profile, which remained unchanged. The reduction of inter-chain disulfide bonds and the corresponding formation of free heavy and light chains was confirmed by SDS-PAGE. The excess of DTT used for reduction was quenched with IAA prior to SDS-PAGE (Fig. S-1). The mild, non-denaturing conditions that were utilized for ion-exchange chromatography appeared not to be sufficient to break the hydrophobic and electrostatic interactions between the heavy and light chains of the antibody. Size-exclusion chromatography of intact and reduced trastuzumab supported this assumption, since both samples showed a single peak corresponding to the intact antibody (Fig. S-2). A similar observation was reported when hydrophobic interaction chromatography of intact and reduced antibodies gave a single peak corresponding to the intact antibody [11]. This lead us to the conclusion that heavy and light chains of antibodies cannot be chromatographically separated despite inter-chain disulfide bond reduction using native chromatographic methods such as ion-exchange, size-exclusion, or hydrophobic interaction chromatography, unless denaturing conditions are applied. They can be separated by reversed-phase liquid chromatography (RPLC) in the presence of organic modifier, elevated temperature, and acidic mobile phase additives [11, 33, 34]. However, RPLC is not suitable to separate charge variant of mAbs.

### Charge variant separation at the subunit level after limited proteolytic cleavage

**Fig. 2:**
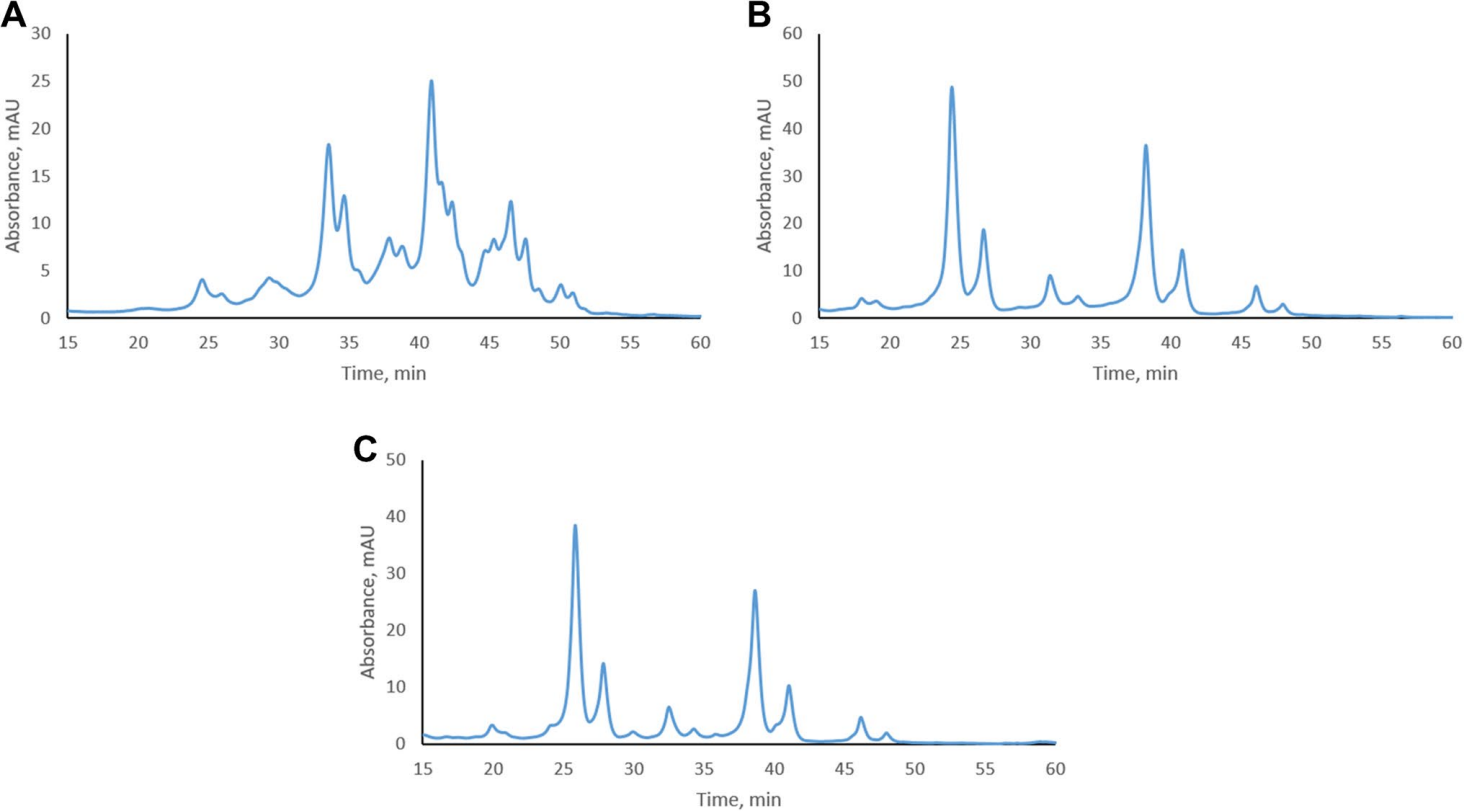
Cation-exchange profile of **A** 3 weeks stressed trastuzumab after IdeS digestion and **B** 3 weeks stressed trastuzumab after GingisKHAN digestion, **C** 3 weeks stressed trastuzumab after papain digestion. UV absorbance was measured at 280 nm

A new generation of proteases such as IdeS and GingisKHAN open alternative options for generating antibody subunits [35–38]. IdeS is known to cleave antibodies below the hinge region to form F(ab)2 and Fc/2 subunits. In trastuzumab, cleavage occurs between two glycine residues that are located at positions 239 and 240 in the heavy chain. Digestion with GingisKHAN generates Fab and Fc subunits through cleavage above the hinge region between lysine and threonine at positions 225 and 226 in the heavy chain. The cation-exchange profile of 3 weeks stressed trastuzumab after IdeS digestion (Fig. 2A) was still complex and similar to that of intact trastuzumab [20]. The reason for this is probably that the F(ab)_2_ fragment generated after IdeS cleavage consists of two Fab domains that are linked by disulfide bonds. As modifications may occur in either one or both Fab domains, this increases the number of possible combinations when two or more modifications are present in the Fab region. A similar issue was encountered when separating charge variants of stressed intact trastuzumab [20]. The number of possible combinations was reduced when the charge variants were analyzed at the Fab level after GingisKHAN digestion. Digestion with GingisKHAN resulted in a simpler chromatographic profile with baseline separated peaks as shown in Fig. 2B.

Papain is a widely used protease known to digest IgG class antibodies into smaller subunits [39]. The first cleavage occurs above the hinge region between the histidine-threonine bond at positions 227 and 228 in the heavy chain of trastuzumab. A second cleavage occurs below the hinge region between the glutamic acid-leucine bond at positions 236 and 237 of the heavy chain. Papain’s cleavage site specificity is pH-dependent, mostly cleaving below the hinge region under slightly acidic conditions [40]. Such variability in cleavage sites may result in a mixture of fragments (Fab and F(ab)_2_), increasing sample complexity. In our case with digestion at pH 7, the papain digested sample showed an almost identical chromatographic profile to the GingisKHAN digested sample (Fig. 2C). Papain required a longer digestion time than GingisKHAN (18 h versus 2 h). Obtaining complete digestion is important because an incomplete digestion may lead to a mixed profile of intact and Fab level trastuzumab. While producing similar profiles, papain is considerably cheaper than GingisKHAN, which favors its use when there is a need to digest larger amounts of mAbs for further analysis.

The Fc domain of trastuzumab did not bind to the cation-exchange column and eluted as a flow-through peak (Fig. S-3). With a calculated pI of 6.74 (https://www.protpi.ch/Calculator/ProteinTool), the Fc domain is negatively charged at the starting pH of 8 of the cation-exchange separation. The same applies to the Fc and Fc/2 fragments that are generated after GingisKHAN and IdeS digestion, respectively. Consequently, pH gradient CEX allowed obtaining F(ab)_2_ or Fab level charge heterogeneity profiles without interference from the Fc domain.

### MS characterization of charge variants separated at the Fab level

**Fig. 3:**
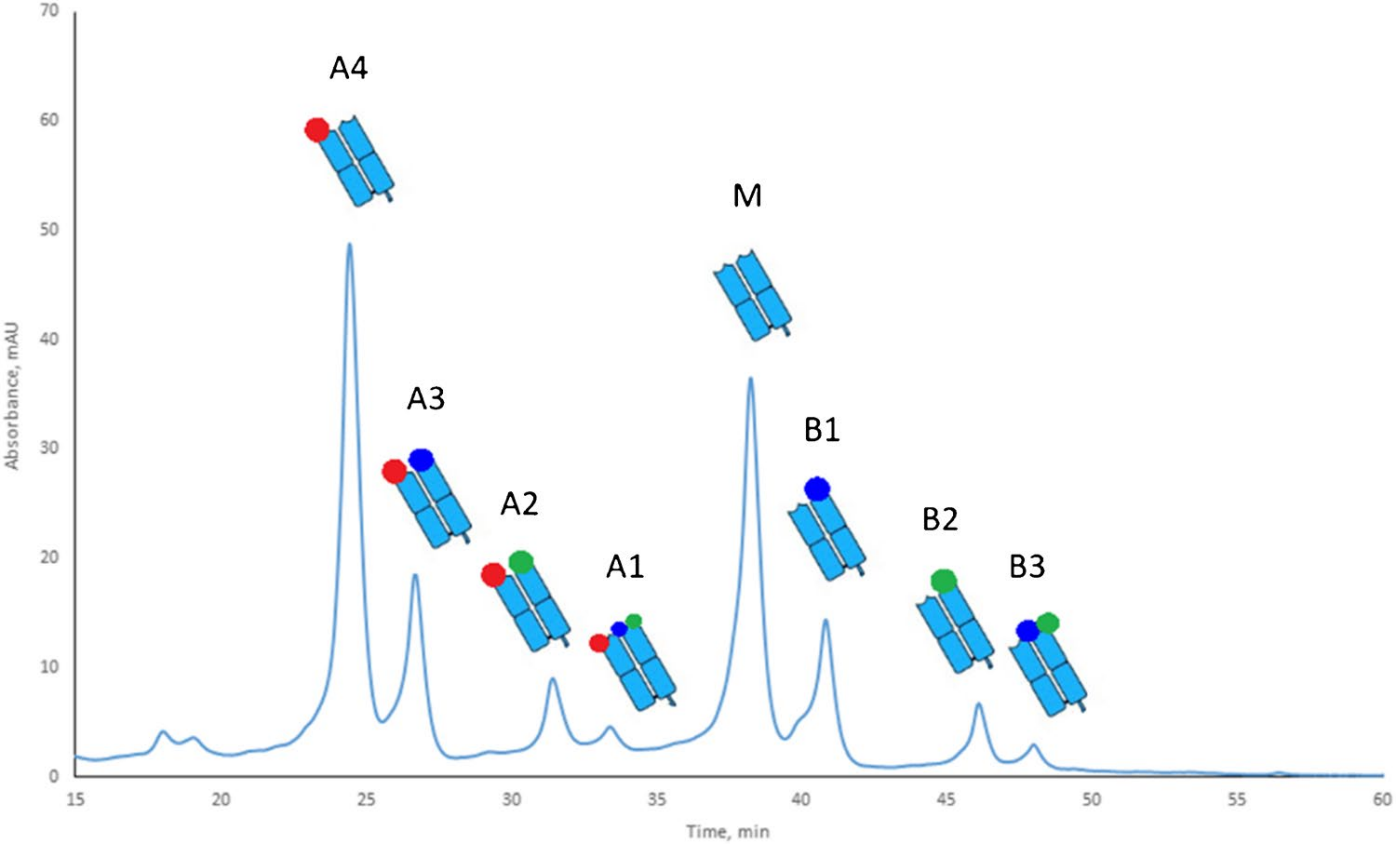
Assignment of charge variants of 3 weeks stress trastuzumab separated at the Fab level after GingisKHAN digestion. The red dot indicates deamidation in the light chain at Asn-30, the blue dot indicates isomerization in the heavy chain at Asp-102, and the green dot indicates N-terminal pyroglutamate formation in the heavy chain

Peptide mapping of the charge variants of 3 weeks stressed trastuzumab separated at the Fab level after GingisKHAN digestion was performed after fractionation on the cation-exchange column (Fig. 3). The purity of fractions was assessed by SDS-PAGE (Fig. S-4). The assignment of modifications was performed by chromatographic separation of native (non-modified) and modified peptides followed by MS/MS fragmentation (Figs. S-5–S-8). Deamidation of asparagine at position 30 in the light chain (Lc-Asn-30), aspartic acid isomerization at position 102 in the heavy chain (Hc-Asp-102), and N-terminal pyroglutamate formation in the heavy chain of trastuzumab were detected after peptide mapping of the collected fractions. According to the results of peptide mapping, the average sequence coverage among the different fractions was 71% for the light chain and 64% for the heavy chain. Sequence coverage was not complete, since very long peptides (more than 30 amino acids) and very short peptides (2–4 amino acids) were not detected. However, all modifications found in this study agree with those from previously published studies on trastuzumab [19, 20, 41]. The modification-dependent order of elution of the Fab level charge variants was the same as that of the intact antibody (Fig. 3). However, separation at the Fab level resulted in baseline separation of charge variants. A few combinations of modifications in fractions A1, A2, and B3 were found after Fab level separation, while we were unable to assign them after separation at the intact mAb level.

**Fig. 4:**
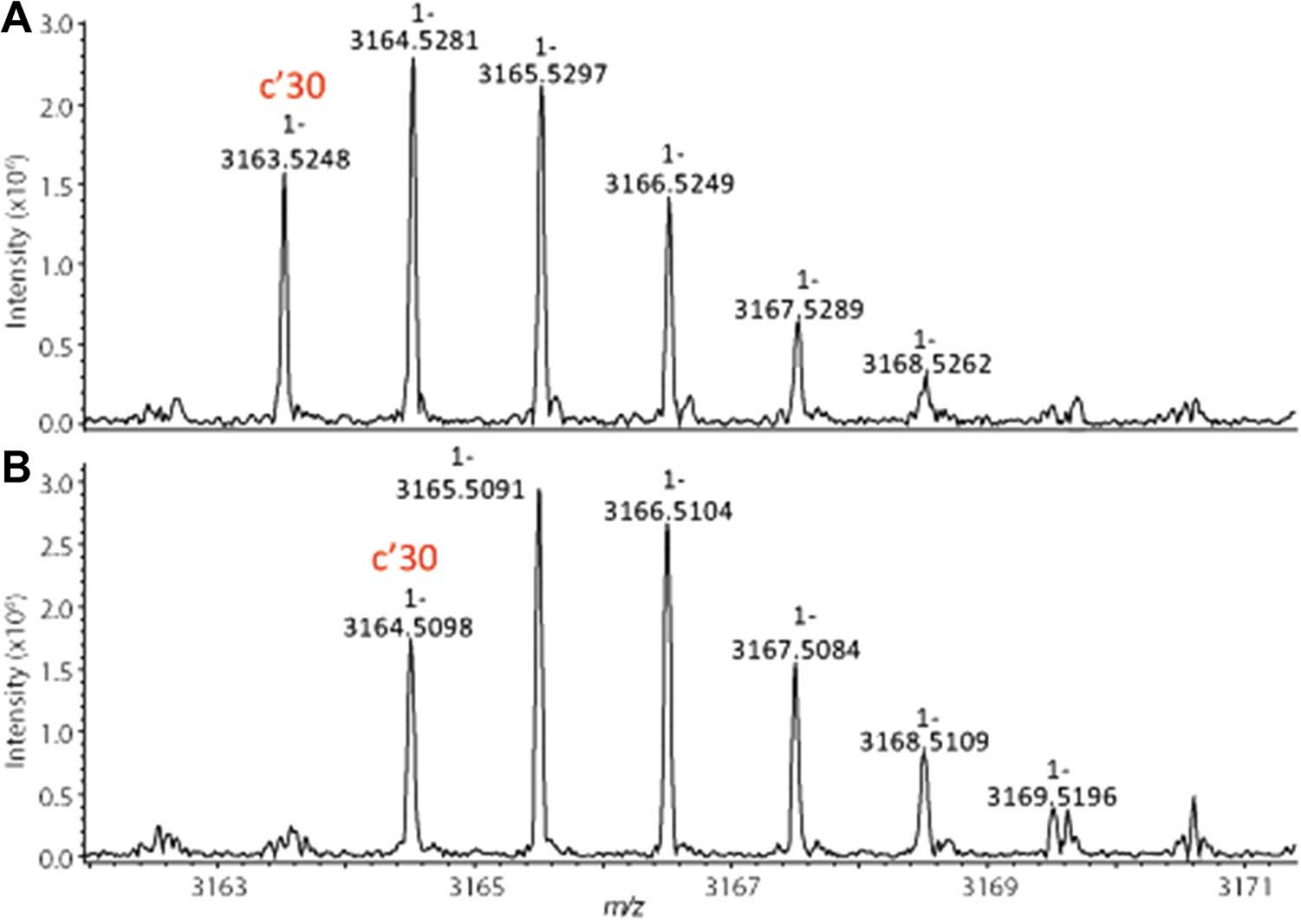
Enlargement of MALDI-ISD FT-ICR mass spectra obtained from a non-deamidated (**A**) and a deamidated (**B**) sample (fractions M and A4 in Fig. 3). The measured c’30 ions depicted in panels **A** and **B** correspond to the light chain fragments DIQMTQSPSSLSASVGDRVTITCRASQDVN (theoretical m/z 3163.5117) and DIQMTQSPSSLSASVGDRVTITCRASQDVD (theoretical m/z 3164.5196), respectively

MALDI-ISD MS was previously reported as a complementary technique to study the N- and C-terminal amino acid sequences of monoclonal antibodies and relevant post-translational modifications [32, 42–46]. To evaluate the potential of this technique, Fab fractions from the GingisKHAN digestion (see Fig. 3) were analyzed by MALDI-ISD FT-ICR MS. Lc-Asn-30 deamidation in fraction A4 was confirmed by comparison of the c’30 fragment ions upon analysis of fractions A4 and M based on a mass increase of 1 Da, as indicated by the corresponding shift in the isotopic pattern (Fig. 4).

## Discussion

In this study, we explored approaches to separate charge variants of mAbs at the subunit level on the example of trastuzumab. Separation of charge variants after reduction of inter-chain disulfide bonds was not possible due to intra-molecular interactions between the light and heavy chains of trastuzumab leading to strongly overlapping peaks and a complex chromatographic profile. The use of IdeS did not resolve this issue, since the separation at the F(ab)_2_ level resulted in a similarly complex chromatogram with overlapping peaks. On the contrary, separation of charge variants with improved resolution at the Fab level was obtained after GingisKHAN and papain digestion. The application of a pH gradient starting at pH 8 has the additional advantage of eliminating the Fc domain in the flow-through. This approach may be applied to charge variant characterization at the Fab level of other antibodies, since most mAbs have a basic pI [47]. Separating trastuzumab charge variants with pH gradient buffers showed that the pH value at which charge variants elute from the column correlates with their pI value as determined by isoelectric focusing [22]. Separation at the Fab level opens opportunities for the isolation of purified charge variants to study the monovalent binding of Fab fragments to the target antigen. The latter may be of interest, particularly for bispecific antibodies where charge variants corresponding to each Fab can be isolated and their binding affinity to the specific antigen can be measured.

Charge variant analysis by coupling ion-exchange chromatography to MS using volatile buffers has recently emerged as an approach to study charge heterogeneity of mAbs at the intact protein level [24, 25]. However, so far, this approach relies solely on an accurate mass measurement and does not allow to localize modification sites unless it is followed by MS/MS fragmentation (top-down MS). While new developments in mass spectrometry may alleviate this issue, it is currently not possible to obtain sufficient sequence coverage of mAbs by top-down MS at the intact protein level. Middle-down MS at the subunit level has more potential in this respect, since MS/MS fragmentation is more feasible than fragmenting an intact 150 kDa antibody. Our initial data show that middle-down MALDI-ISD FT-ICR MS at the Fab level allows confirming the deamidation of Lc-Asn-30. Middle-down MS did, however, not allow to assign all of the observed modifications indicating a need for further improvements of this approach.

## Supplementary Information

Below is the link to the electronic supplementary material.

Supplementary file1 (DOCX 1916 KB)
