# Supplementary material for: Revealing charge heterogeneity of stressed trastuzumab at the subunit level
Source: Anal Bioanal Chem. 2023 Jan 25;415(8):1505–13. doi: 10.1007/s00216-023-04547-4 (PMC9974696; doi:10.1007/s00216-023-04547-4)
Supplement: Supplementary file 1 — Supplementary file1 (DOCX 1916 KB) [file 216_2023_4547_MOESM1_ESM.docx]

**Revealing charge heterogeneity of stressed trastuzumab at the subunit level**

**Baubek Spanov^a^, Bas Baartmans^a^, Oladapo Olaleye^a^, Simone Nicolardi^b^, Natalia Govorukhina^a^, Manfred Wuhrer^b^, Nico C. van de Merbel^a, c^, Rainer Bischoff^a*^**

*^a^* *Department of Analytical Biochemistry, Groningen Research Institute of Pharmacy, University of Groningen, A Deusinglaan 1, 9713 AV Groningen, The Netherlands*

*^b^ Center for Proteomics and Metabolomics, Leiden University Medical Center, 2333 ZA Leiden, The Netherlands*

*^c^ Bioanalytical Laboratory, ICON, Amerikaweg 18, 9407 TK Assen, The Netherlands*

**Corresponding Author: Rainer Bischoff** − *Department of Analytical Biochemistry, Groningen Research Institute of Pharmacy, University of Groningen, 9713 AV Groningen, The Netherlands; orcid.org/0000-0001-9849-0121; Email: r.p.h.bischoff@rug.nl*


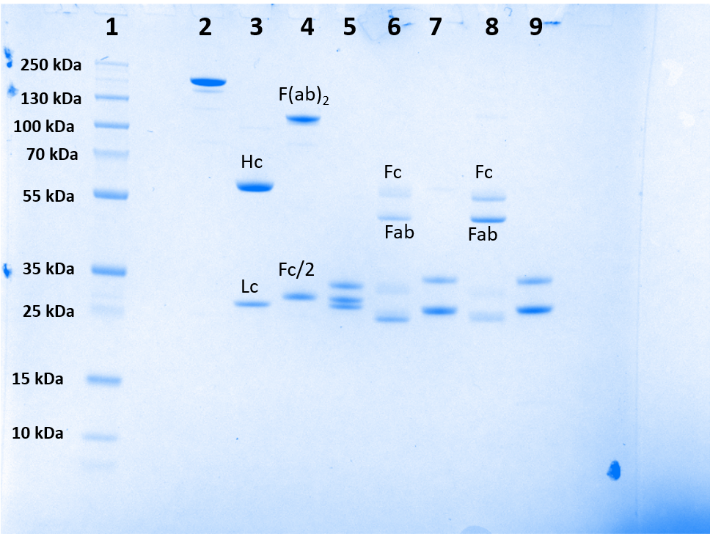


**Figure S-1**. SDS-PAGE of trastuzumab subunits. Line-1: prestained protein markers; line-2: intact trastuzumab; line-3: reduced trastuzumab; line-4: IdeS digested trastuzumab; line-5: IdeS digestion followed by reduction; line-6: GingisKHAN digested trastuzumab; line-7: GingisKHAN digestion followed by reduction; line-8: papain digested trastuzumab; line-9: papin digestion followed by reduction.


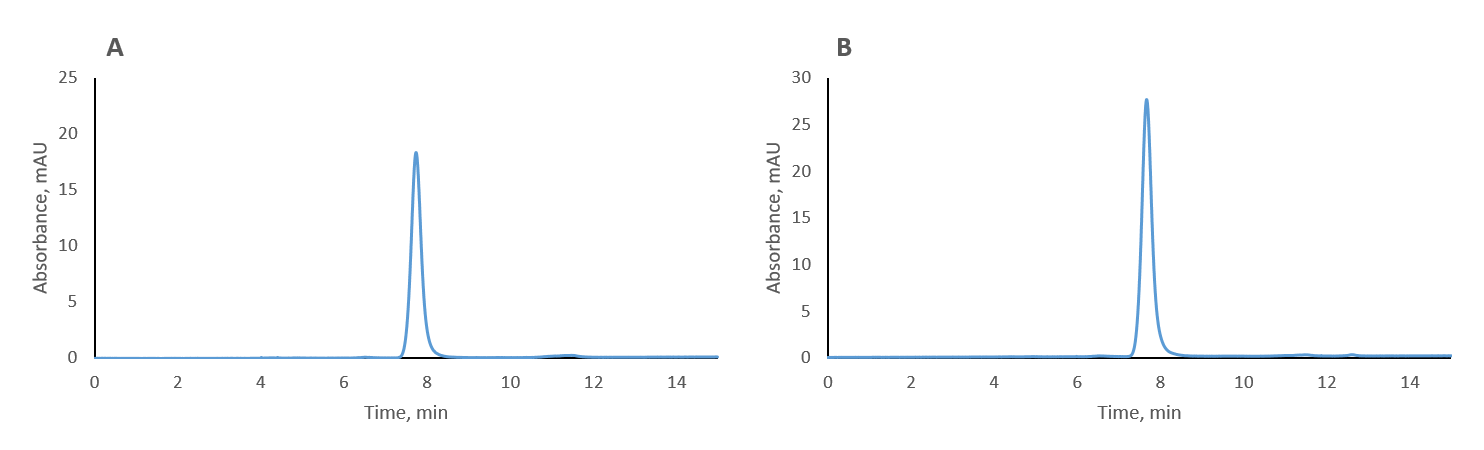


**Figure S-2**. Size-exclusion chromatography of (A) intact and (B) reduced trastuzumab. An Agilent 1200 HPLC system was used for the SEC analysis. 20 µL of 1 mg/mL protein samples were injected into the TSKgel G2000SWXL column operated at room temperature. The mobile phase consisted of 100 mM phosphate buffer pH 6.7. The flow was set to 0.5 mL/min. UV absorbance was measured at 280 nm.


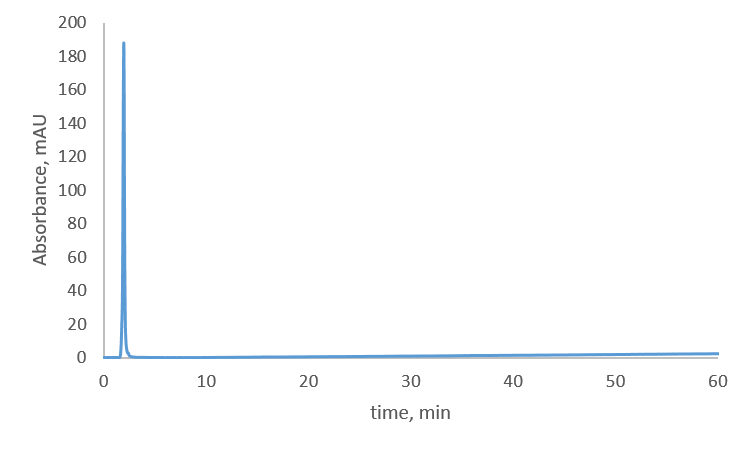


**Figure S-3**. Elution of the Fc domain of trastuzumab (after SEC) in the flow-through fraction of the pH gradient CEX. UV absorbance was measured at 280 nm.


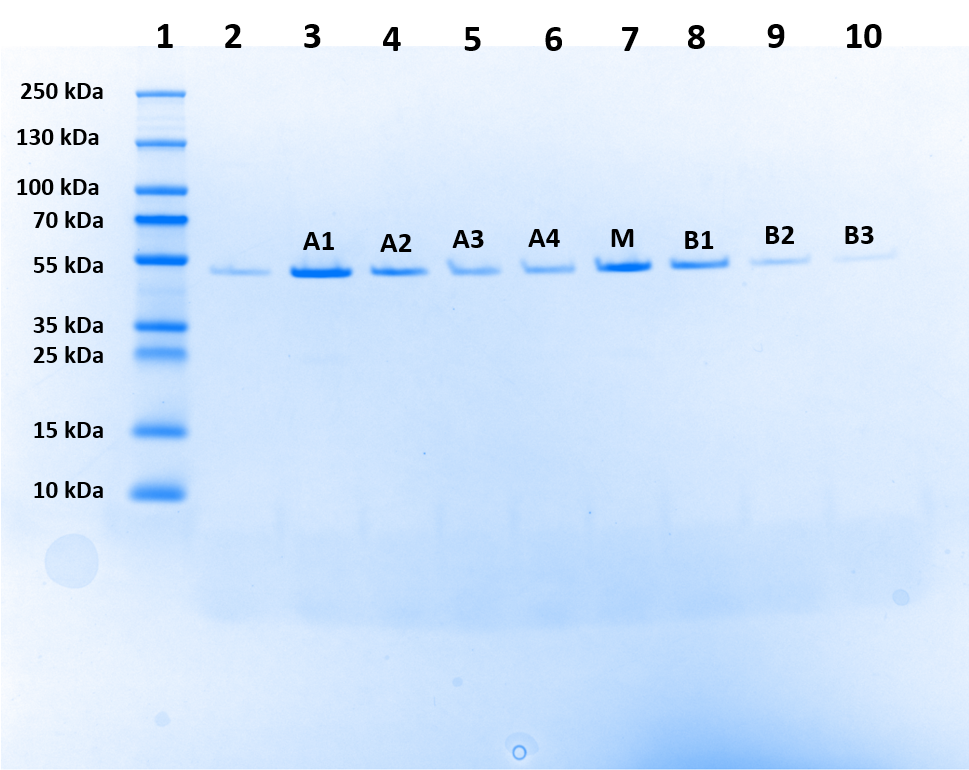


**Figure S-4**. SDS-PAGE of fractions collected from the cation-exchange column after GingisKHAN digestion as shown in **Figure 3**.


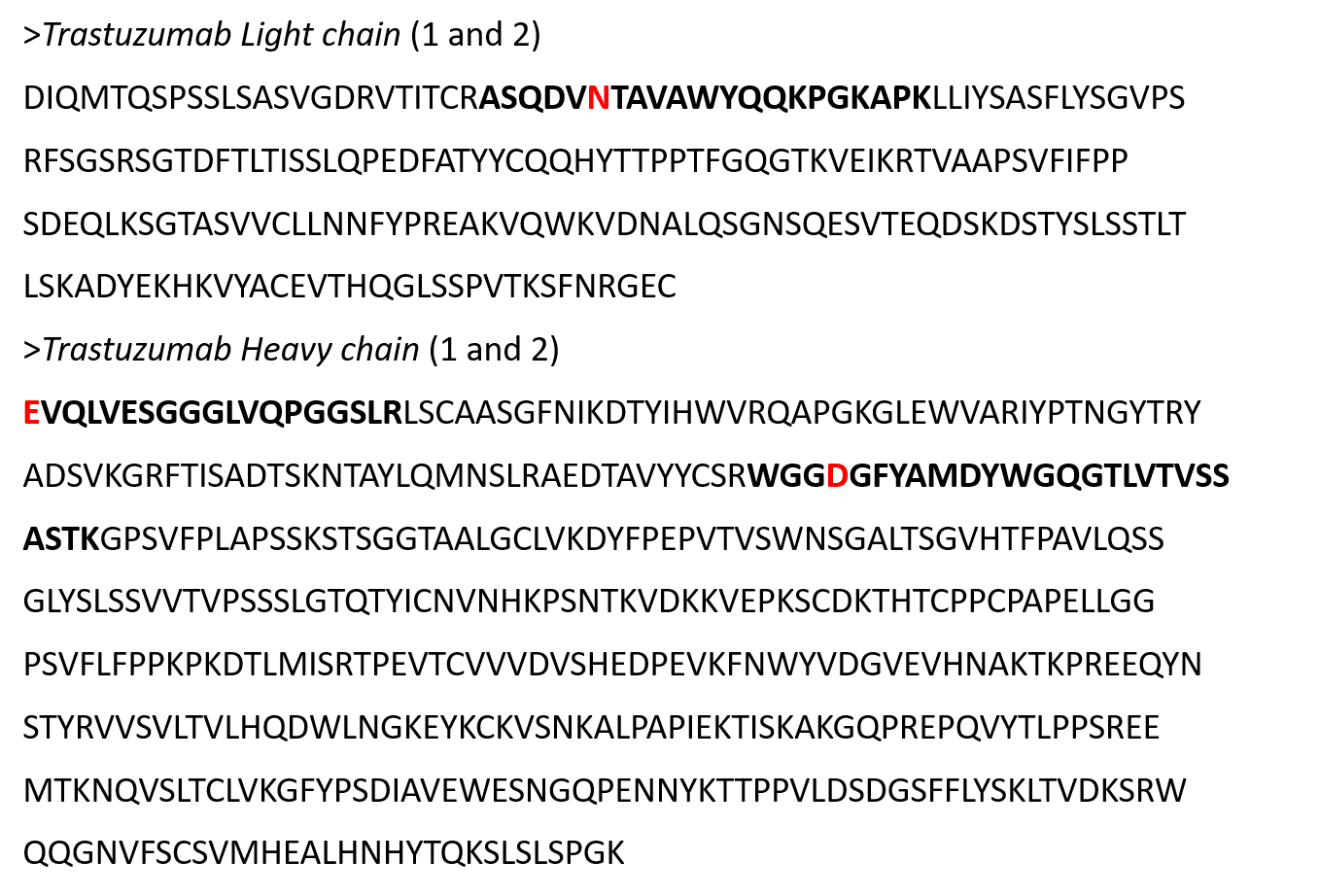


**Figure S-5**. Amino acid sequence of trastuzumab (https://go.drugbank.com/drugs/DB00072). Peptides used for identification and quantification of modifications are shown in bold. Amino acids that were shown to be modified are shown in red.


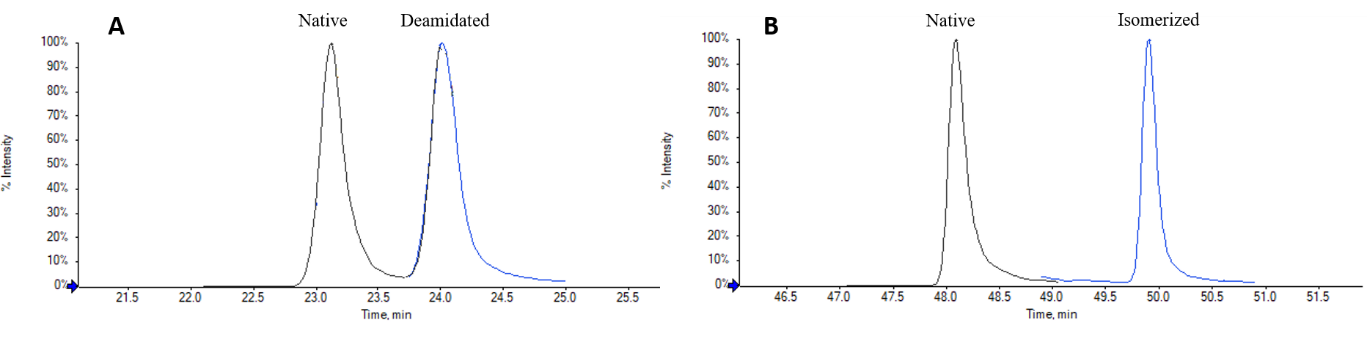


**Figure S-6**. LC-MS chromatograms of peptides highlighted in **Figure S-5**. (A) Native and deamidated peptides at the Lc-Asn-30 position; (B) Native and isomerized peptides at the Hc-Asp-102 position.


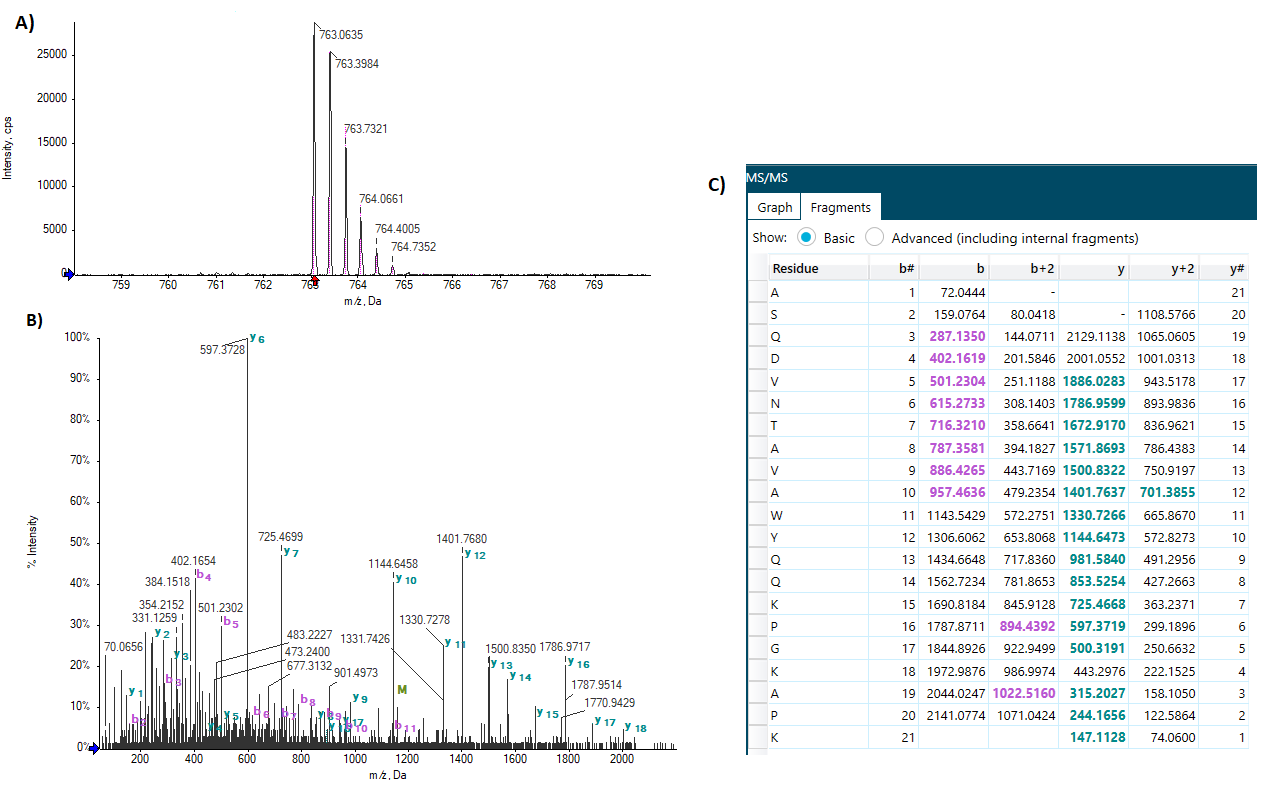


**Figure S-7. A)** Precursor mass selection followed by MS/MS fragmentation of the native peptide (ASQDVNTAVAWYQQKPGKAPK) from the light chain of trastuzumab as indicated in Figures S-5 and S-6. The theoretical monoisotopic m/z of the triply-charged peptide is 763.0658 (+3), the measured m/z was 763.0694 (+3). The precursor ion 763.0694 (+3) was further selected for MS/MS fragmentation. **B)** MS/MS fragments from m/z 763.0694 (+3). **C)** Identified y and b ions are colored.


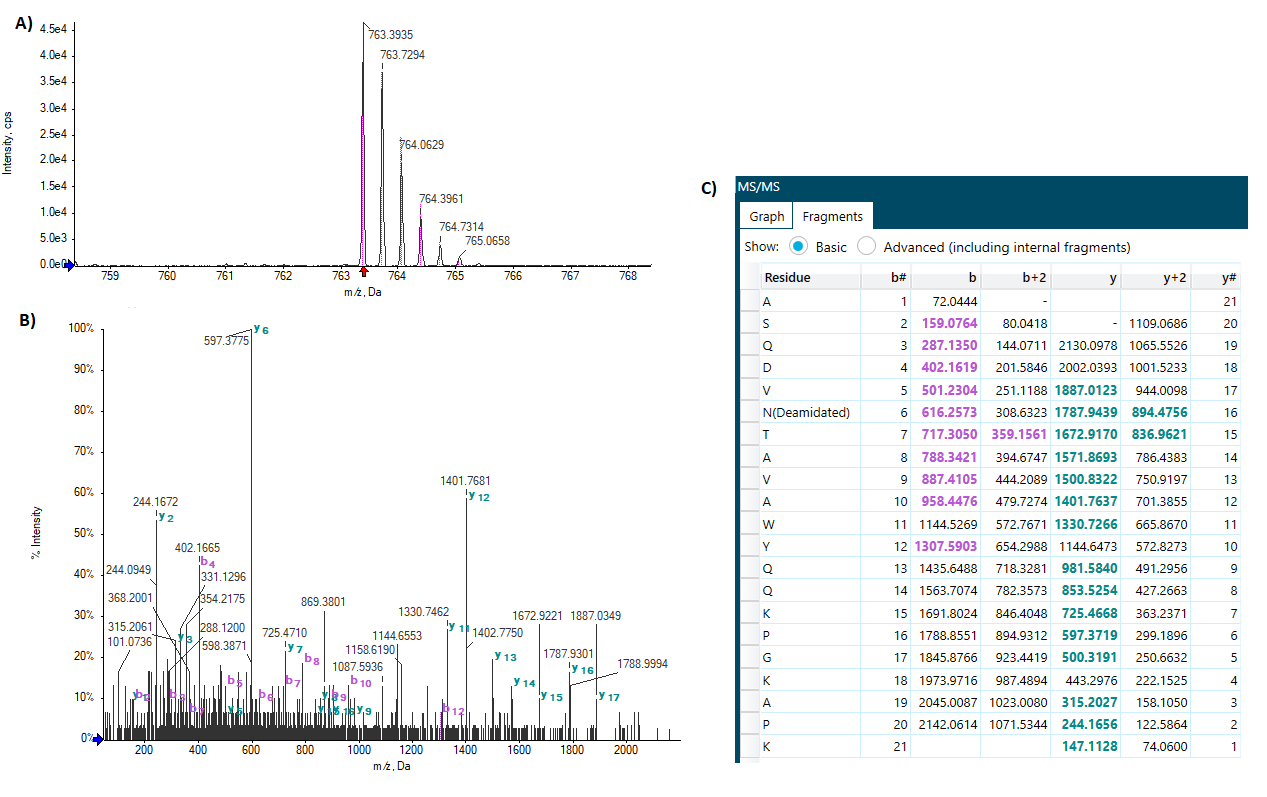


**Figure S-8. A)** Precursor mass selection followed by MS/MS fragmentation of the deamidated peptide (ASQDV**N**TAVAWYQQKPGKAPK) from the light chain of trastuzumab as indicated in Figures S-5 and S-6. The theoretical monoisotopic m/z of the triple charged peptide is 763.3938 (+3), the measured m/z was 763.3935 (+3). The precursor ion 763.3935 (+3) was further selected for MS/MS fragmentation. **B)** MS/MS fragments from m/z 763.3935 (+3). **C)** Identified y and b ions are colored.
